# Supplementary material for: Structural pharmacology of SV2A reveals an allosteric modulation mechanism in the major facilitator superfamily
Source: Nat Commun. 2025 Nov 28;16:10748. doi: 10.1038/s41467-025-65781-1 (PMC12663137; doi:10.1038/s41467-025-65781-1)
Supplement: Supplementary file 1 — Supplementary Information [file 41467_2025_65781_MOESM1_ESM.pdf]

Supplementary Information

Structural pharmacology of SV2A reveals an allosteric modulation mechanism in the major facilitator superfamily

Shabareesh Pidathala[1], Xiao Chen[1], Yaxin Dai[1], Long N. Nguyen[2], Christoph Gorgulla[1], Yiming Niu[3], Fangyu Liu[4]*, and Chia-Hsueh Lee[1]*

[1]Department of Structural Biology, St. Jude Children's Research Hospital, Memphis, TN, USA.

[2]Department of Biochemistry, Yong Loo Lin School of Medicine, National University of Singapore, Singapore 119228.

[3]Laboratory of Chromosome and Cell Biology, The Rockefeller University, New York, NY, USA.

[4]Department of Pharmacology, UT Southwestern Medical Center, Dallas, TX, USA

*Correspondence: fangyu.liu@utsouthwestern.edu and chiahsueh.Lee@stjude.org

The PDF file includes:

Supplementary Figure 1–7

Supplementary Table 1

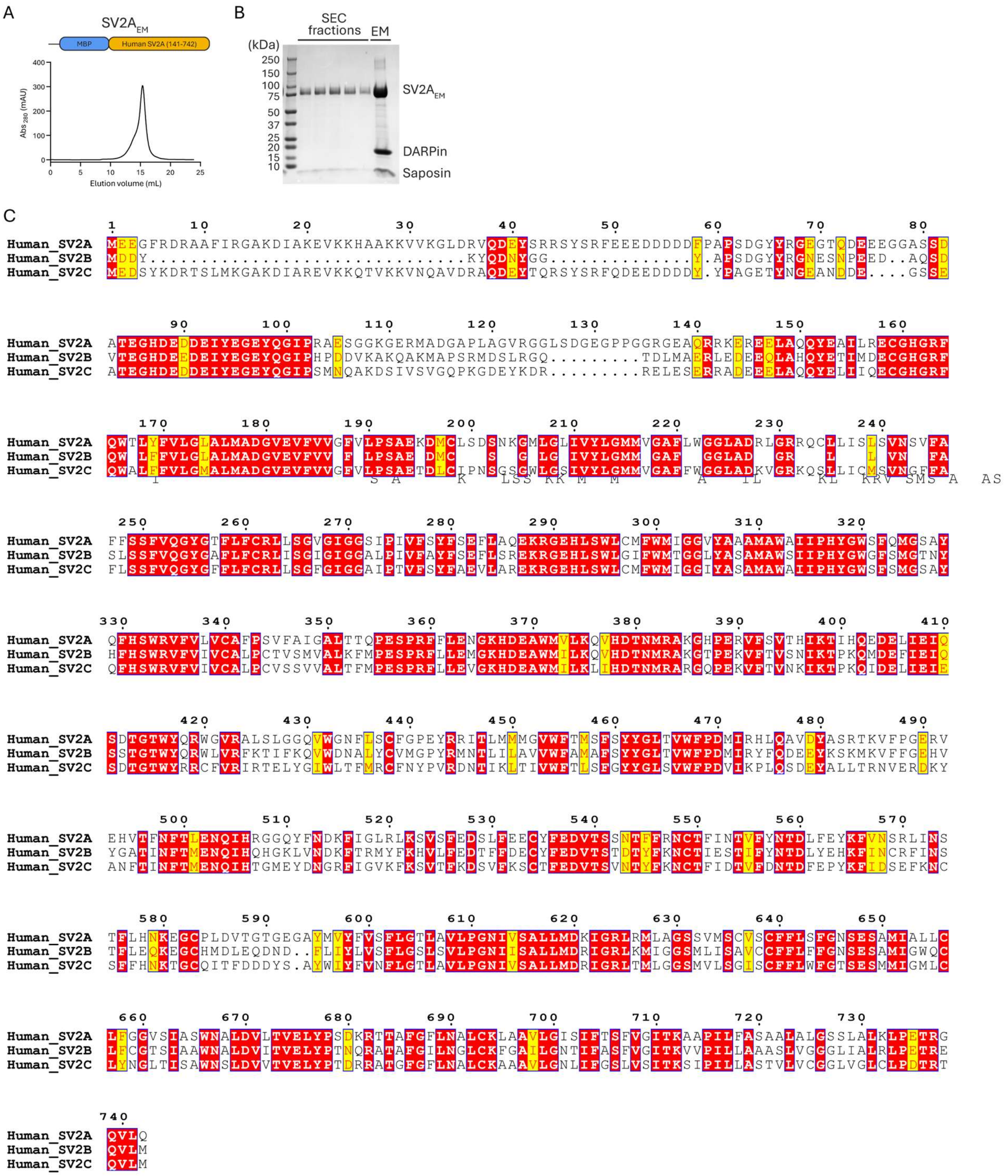

Supplementary Fig. 1: Construct design and sequence alignment of SV2 proteins.

A Schematic showing the design of human SV2A$_{EM}$ construct and a representative SEC profile for purification of SV2A$_{EM}$. **B** SDS-PAGE analysis of purified SV2A$_{EM}$ protein. **C** Sequence alignment of three isoforms of human SV2s.

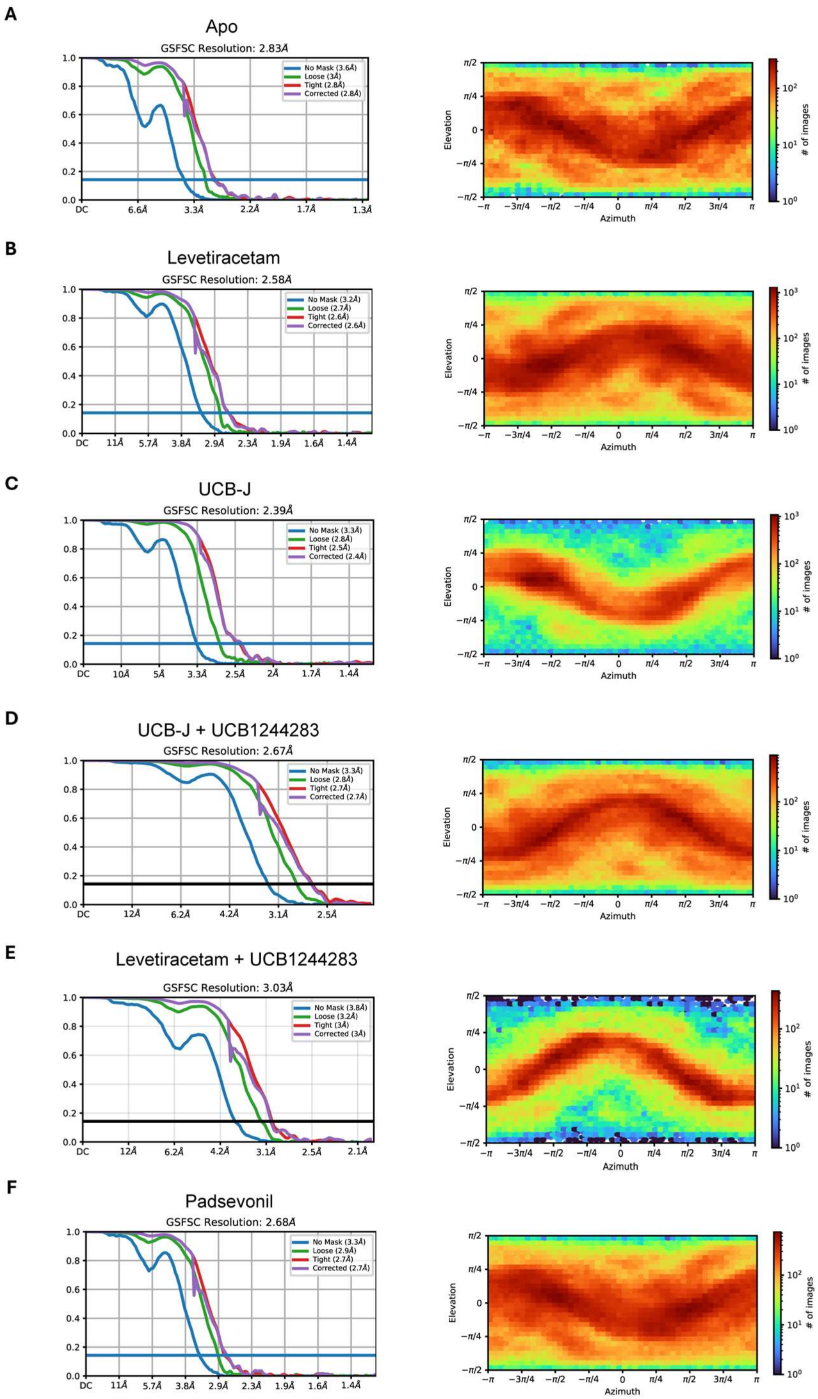

Supplementary Fig. 2: Cryo-EM analyses of SV2A$_{EM}$ structures.

Fourier-shell correlation (FSC) curves between two half-maps of **A** SV2$_{EM}$ apo, **B** levetiracetam, **C** UCB-J, **D** UCB-J+UCB1244283, **E** levetiracetam+UCB1244283, and **F** padsevonil bound states along with their corresponding angular distribution of particles used in final 3D reconstructions.

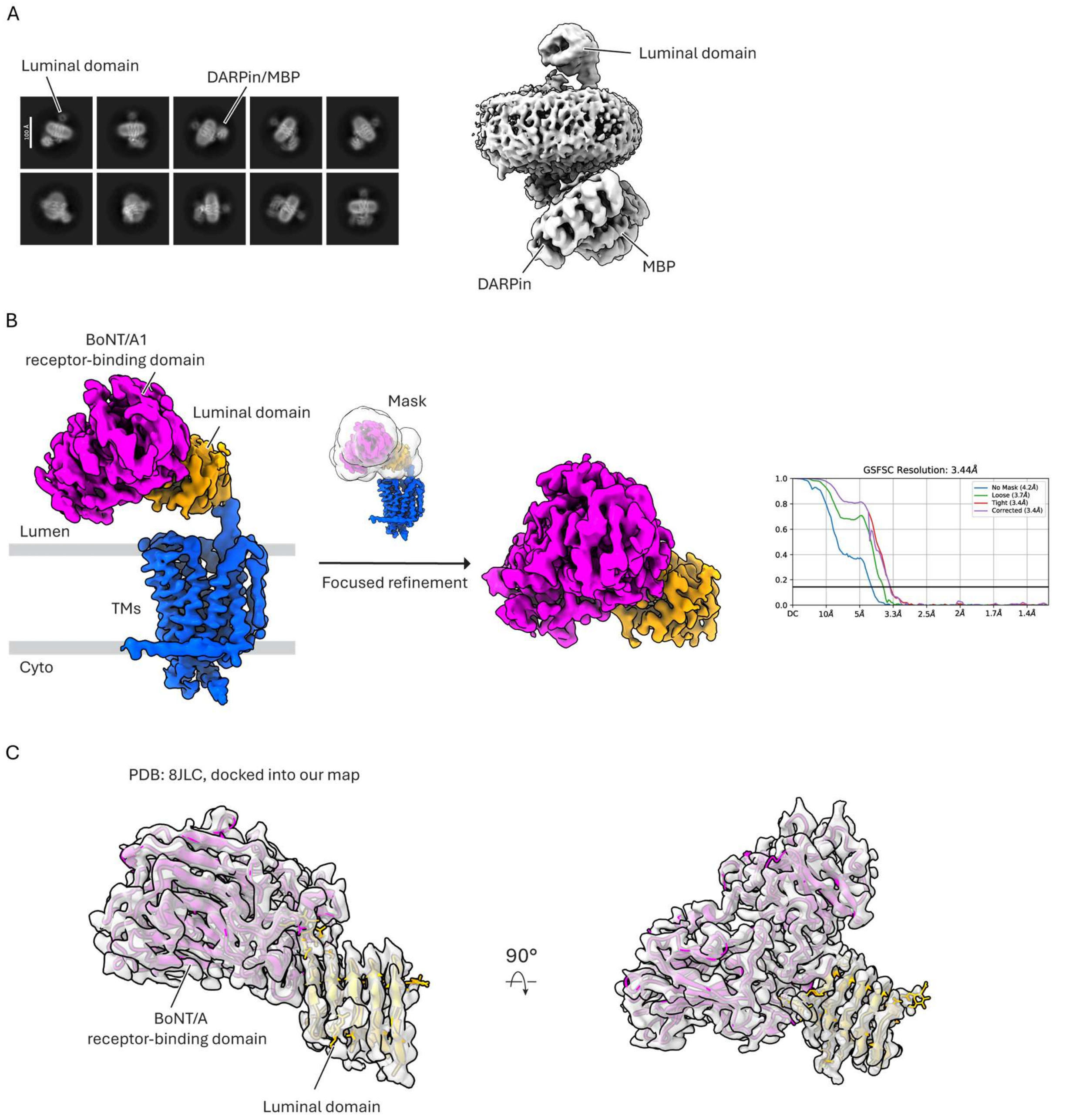

Supplementary Fig. 3: Structural investigation on the luminal domain of $SV2A_{EM}$.

A Left, representative 2D classes of $SV2A_{EM}$, showing density corresponding to the luminal domain. Right, unsharpened map highlighting the structural features of the luminal domain. **B** Structure of $SV2A_{EM}$ in complex with the receptor binding domain of BONT/A1. **C** Comparison between the SV2A-toxin structures from the current study and a previous report. The reported structure fits well into our density map, indicating a high degree of structural similarity.

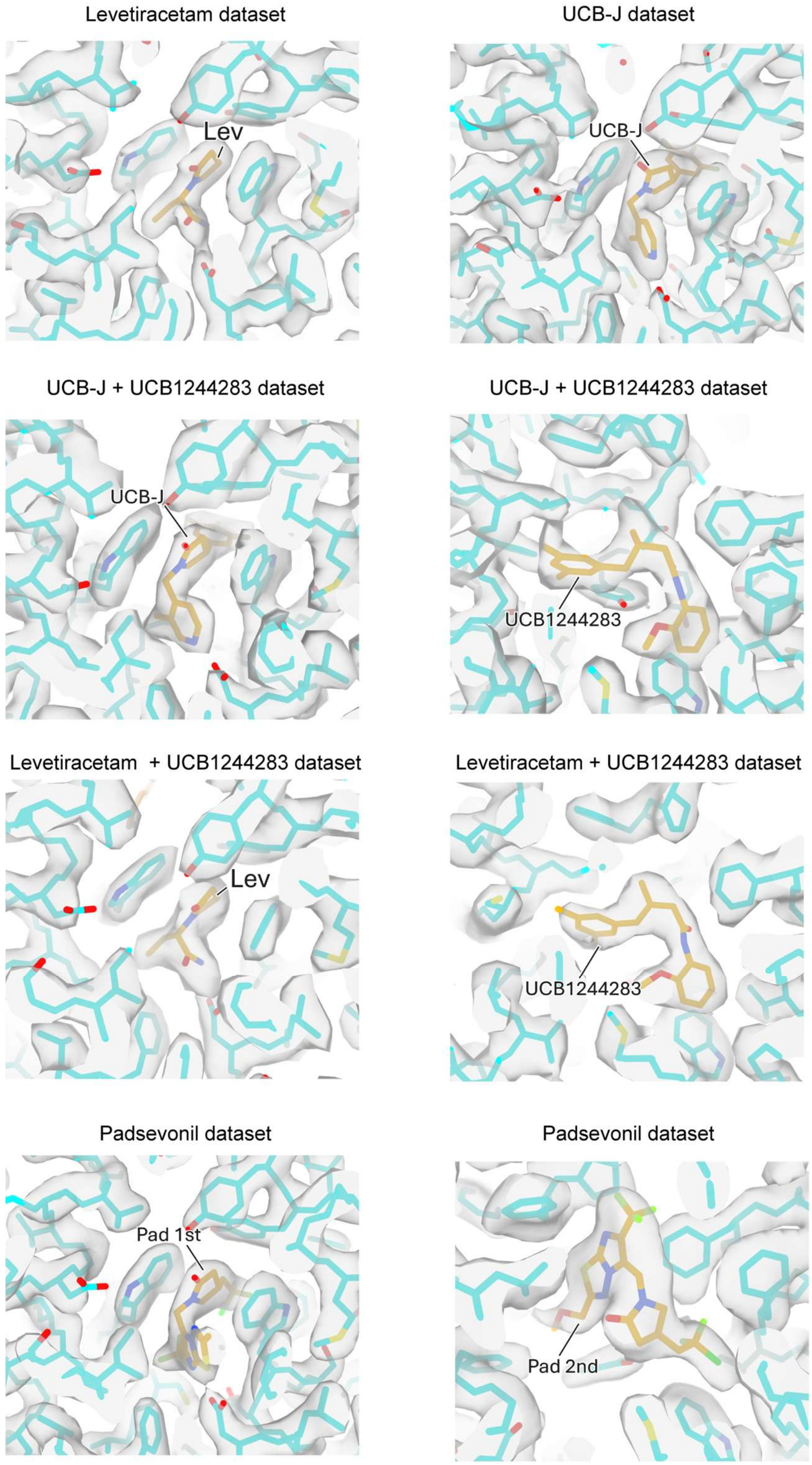

Supplementary Fig. 4: Cryo-EM density of ligands.

Cryo-EM density of SV2A in complex with different ligands, focusing on the ligands and their surrounding residues.

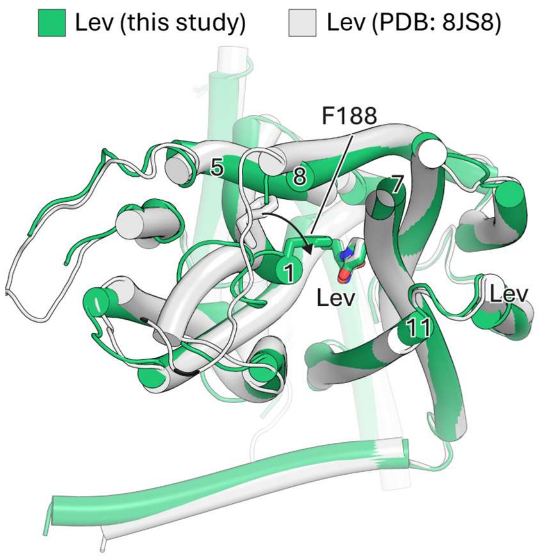

Supplementary Fig. 5: Comparison of SV2A-Lev structures.

Overlay of our SV2A-Lev structure (green) with the SV2A-Lev structure in complex with Botulinum toxin A2 (PDB: 8JS8) (grey). The TM1 helix in our structure undergoes a pronounced displacement towards the vestibule which is not observed in the reported structure.

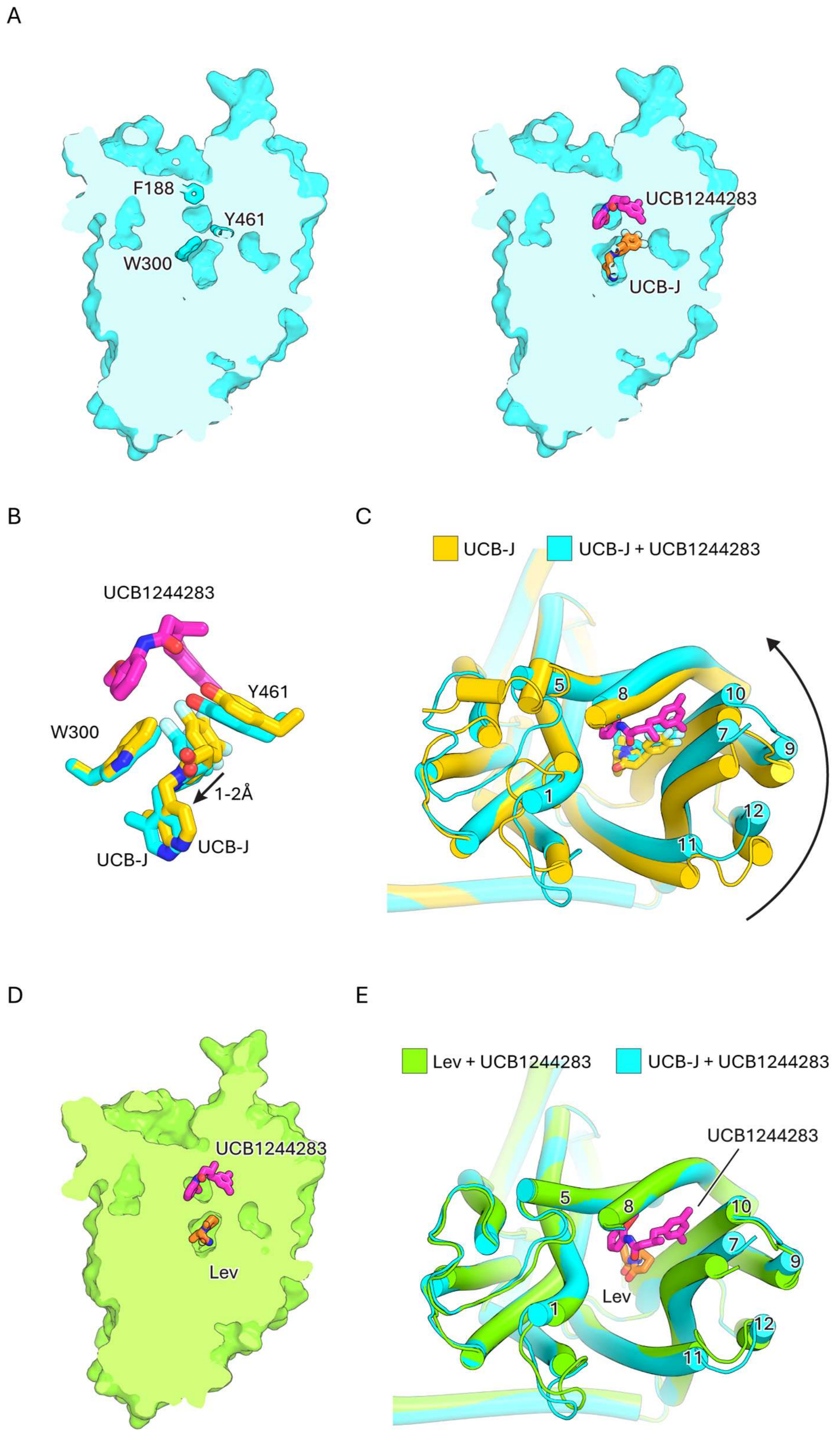

A
F188
Y461
W300
UCB1244283
UCB-J
B
UCB1244283
Y461
W300
1-2Å
UCB-J
UCB-J
C
UCB-J
UCB-J + UCB1244283
5
8
10
7
9
1
12
11
D
UCB1244283
Lev
E
Lev + UCB1244283
UCB-J + UCB1244283
UCB1244283
5
8
10
7
9
Lev
1
12
11

Supplementary Fig. 6: Comparison of SV2A-UCB-J, SV2A-UCB-J/UCB1244283 and SV2A-Lev/UCB1244283 structures.

A Cut-open view of SV2A with UCB1244283 (magenta) in the allosteric site and UCB-J (orange) in the orthosteric site. An occluded vestibule is formed with residues closing the orthosteric and allosteric sites. **B** Effect of UCB1244283 (magenta) binding on UCB-J in the orthosteric site. UCB-J in the UCB-J/UCB1244283 structure is in cyan, whereas UCB-J in the UCB-J only structure is in yellow. **C** TM helices movement observed in the C-terminal half of SV2A in the UCB-J/UCB1244283 complex (cyan), compared to the UCB-J bound state (yellow). **D** Cut-open view of SV2A with UCB1244283 (magenta) in the allosteric site and levetiracetam (orange) in the orthosteric site. **E** Superposition of levetiracetam/UCB1244283 and UCB-J/UCB1244283 structures.

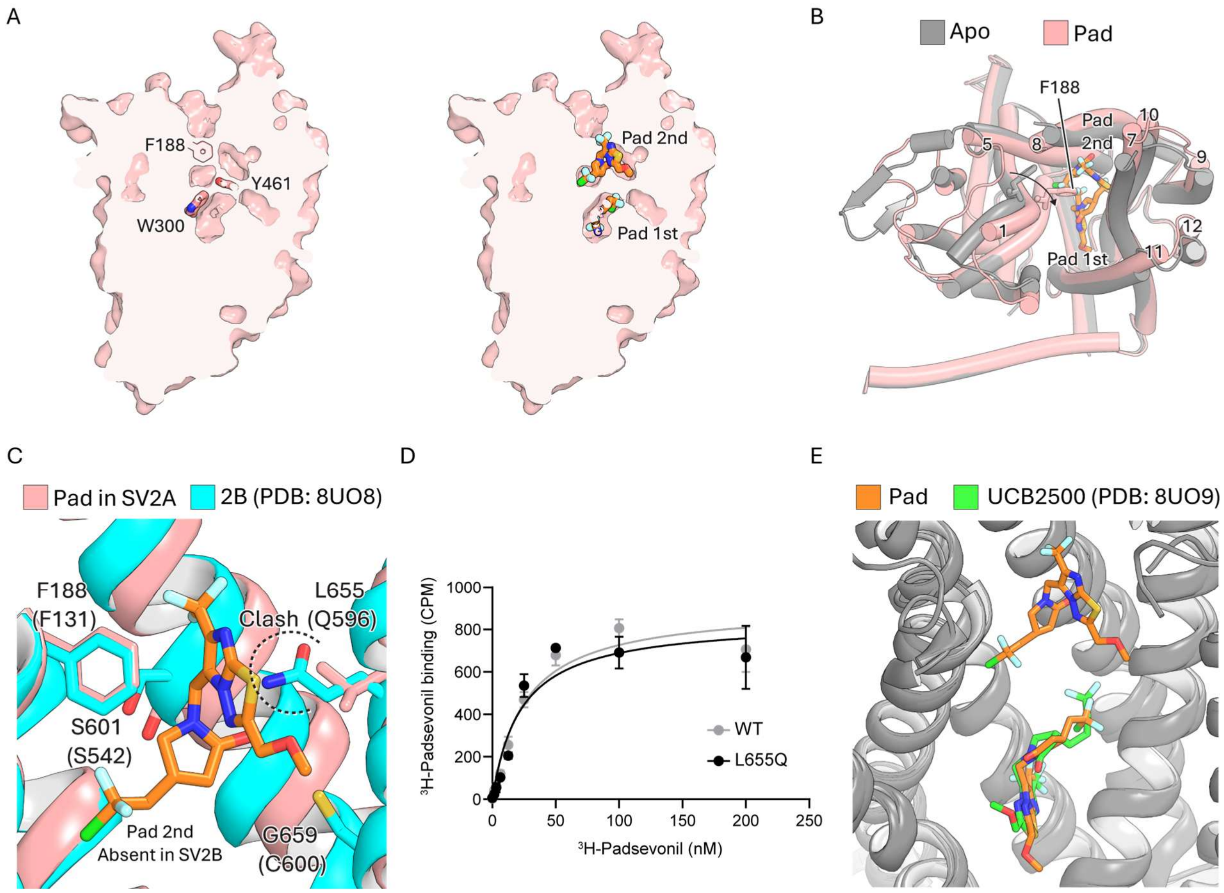

Supplementary Fig. 7: Structural comparison of padsevonil bound SV2A.

A Cut-open view of SV2A with padsevonil (orange) in the allosteric site and orthosteric site. An occluded vestibule is formed with residues closing the orthosteric and allosteric sites. **B** Overlay of SV2A in its padsevonil bound state (salmon) and apo state (grey) showing movement of Phe188 in TM1 towards vestibule leading to the formation of an occluded state. **C** Overlay of allosteric padsevonil site of SV2A (salmon) with that of corresponding site in SV2B (cyan) highlighting residue substitutions at positions that might have precluded the allosteric binding of padsevonil in SV2B. **D** Padsevonil binding to SV2A WT and L655Q. Data are shown as mean ± s.d.; n = 2 biological replicates. **E** Overlay of SV2A bound to padsevonil (orange) and UCB2500 (green) showing the pose of these two ligands and the differences in the alkyl substitutions. Source data for **D** are provided as a Source Data file.

Structure	Apo	Levetiracetam	UCB-J	UCB-J + UCB1244283	Levetiracetam + UCB1244283	Padsevonil
PDB	9OKF	9OKG	9OKH	9OKI	9PRS	9OKJ
EMDB	70562	70563	70564	70565	71812	70566
**Data collection/ processing**						
Magnification	130,000x	130,000x	130,000x	130,000x	130,000x	130,000x
Voltage (kV)	300	300	300	300	300	300
Pixel size (Å)	0.649	0.649	0.649	0.649	0.649	0.649
Defocus range (μm)	1.1–2.1	1.1–2.1	1.1–2.1	1.1–2.1	1.1–2.1	1.1–2.1
Electron exposure (e^-/Å^2)	70.7	70.5	60.0	59.3	60.6	69.0
Symmetry imposed	C1	C1	C1	C1	C1	C1
Initial particles (No.)	~4.8 millions	~11.8 millions	~10.4 millions	~4.9 millions	~6.2 millions	~2.4 millions
Final particles (No.)	271,325	890,424	304,664	546,764	157,236	546,136
Map resolution (Å)	2.83	2.58	2.39	2.67	3.03	2.68
FSC threshold	0.143	0.143	0.143	0.143	0.143	0.143
Map resolution range (Å)	43.3–2.4	29.0–2.2	38.6–2.2	36.4–2.3	44.0–2.6	28.4–2.3
**Refinement**						
Model Resolution (Å)	2.9	2.7	2.7	2.8	3.1	2.8
FSC threshold	0.5	0.5	0.5	0.5	0.5	0.5
Map sharpening B-factor (Å^2)	-85.2	-89.7	-84.0	-124.4	-106.1	-91.3
Model composition						
Non-hydrogen atoms	3753	3735	3718	3789	3778	3797
Protein residues	479	475	472	481	481	481
Ligand	0	1	1	2	2	2
B-factors (Å^2)						
Protein	57.05	41.17	75.58	48.30	56.57	53.79
Ligand	NA	24.15	61.01	40.19	39.52	55.02
R.m.s. deviations						
Bond lengths (Å)	0.002	0.003	0.002	0.002	0.002	0.003
Bond angles (°)	0.468	0.508	0.476	0.457	0.448	0.890
Validation						
MolProbity score	1.00	0.84	1.14	1.20	0.91	0.68
Clashscore	2.27	1.21	3.10	4.12	1.60	0.53
Rotamers outliers (%)	0.77	0.26	0.78	0.00	0.00	0.78
Ramachandran plot (%)						
Favored	99.15	98.72	97.84	98.11	98.95	99.58
Allowed	0.85	1.28	2.16	1.89	1.05	0.42
Outliers	0.00	0.00	0.00	0.00	0.00	0.00

Supplementary Table 1: Cryo-EM data collection, refinement and validation statistics
