# Supplementary material for: Structural pharmacology of SV2A reveals an allosteric modulation mechanism in the major facilitator superfamily
Source: Nat Commun. 2025 Nov 28;16:10748. doi: 10.1038/s41467-025-65781-1 (PMC12663137; doi:10.1038/s41467-025-65781-1)
Supplement: Supplementary file 2 — Reporting Summary [file 41467_2025_65781_MOESM2_ESM.pdf]

nature portfolio

Corresponding author(s): Fangyu Liu, Chia-Hsueh Lee

Last updated by author(s): Sep 17, 2025

Reporting Summary

Nature Portfolio wishes to improve the reproducibility of the work that we publish. This form provides structure for consistency and transparency in reporting. For further information on Nature Portfolio policies, see our Editorial Policies and the Editorial Policy Checklist.

Statistics

For all statistical analyses, confirm that the following items are present in the figure legend, table legend, main text, or Methods section.

n/a	Confirmed	
☐	☒	The exact sample size (n) for each experimental group/condition, given as a discrete number and unit of measurement
☐	☒	A statement on whether measurements were taken from distinct samples or whether the same sample was measured repeatedly
☐	☒	The statistical test(s) used AND whether they are one- or two-sided<br>*Only common tests should be described solely by name; describe more complex techniques in the Methods section.*
☒	☐	A description of all covariates tested
☒	☐	A description of any assumptions or corrections, such as tests of normality and adjustment for multiple comparisons
☐	☒	A full description of the statistical parameters including central tendency (e.g. means) or other basic estimates (e.g. regression coefficient) AND variation (e.g. standard deviation) or associated estimates of uncertainty (e.g. confidence intervals)
☐	☒	For null hypothesis testing, the test statistic (e.g. F, t, r) with confidence intervals, effect sizes, degrees of freedom and P value noted<br>*Give P values as exact values whenever suitable.*
☒	☐	For Bayesian analysis, information on the choice of priors and Markov chain Monte Carlo settings
☒	☐	For hierarchical and complex designs, identification of the appropriate level for tests and full reporting of outcomes
☒	☐	Estimates of effect sizes (e.g. Cohen's d, Pearson's r), indicating how they were calculated

Our web collection on statistics for biologists contains articles on many of the points above.

Software and code

Policy information about availability of computer code

Data collection	EPU 3.1
Data analysis	MotionCor2 1.6.3, cryoSPARC 4, Relion 4, Alphafold 2, COOT 0.9.8, ISOLDE 1.6, Phenix 1.13, MolProbity 4.2, PyMOL 2.5.4, Prism 9.00, Chimera 1.17, ChimeraX 1.6,

For manuscripts utilizing custom algorithms or software that are central to the research but not yet described in published literature, software must be made available to editors and reviewers. We strongly encourage code deposition in a community repository (e.g. GitHub). See the Nature Portfolio guidelines for submitting code & software for further information.

Data

Policy information about availability of data

All manuscripts must include a data availability statement. This statement should provide the following information, where applicable:

- Accession codes, unique identifiers, or web links for publicly available datasets
- A description of any restrictions on data availability
- For clinical datasets or third party data, please ensure that the statement adheres to our policy

Cryo-EM maps and coordinates have been deposited in the EMDB and wwPDB, respectively, with accession numbers: EMD-70562 and PDB 9OKF (apo); EMD-70563 and PDB 9OKG (levetiracetam); EMD-70564 and PDB 9OKH (UCB-J); EMD-70565 and PDB 9OKI (UCB-J +UCB1244283); EMD-71812 and PDB 9PRS (levetiracetam + UCB1244283); EMD-70566 and PDB 9OKJ (padsevonil).

Research involving human participants, their data, or biological material

Policy information about studies with human participants or human data. See also policy information about sex, gender (identity/presentation), and sexual orientation and race, ethnicity and racism.

Reporting on sex and gender	N/A
Reporting on race, ethnicity, or other socially relevant groupings	N/A
Population characteristics	N/A
Recruitment	N/A
Ethics oversight	N/A

Note that full information on the approval of the study protocol must also be provided in the manuscript.

Field-specific reporting

Please select the one below that is the best fit for your research. If you are not sure, read the appropriate sections before making your selection.

[x] Life sciences [ ] Behavioural & social sciences [ ] Ecological, evolutionary & environmental sciences

For a reference copy of the document with all sections, see nature.com/documents/nr-reporting-summary-flat.pdf

Life sciences study design

All studies must disclose on these points even when the disclosure is negative.

Sample size	Statistical methods were not used to determine sample size. The data size for cryo-EM experiments was determined by the availability of microscope time and the particle density on the grids. Sufficient cryo-EM data were collected to achieve the reported resolution of 3D reconstructions. For functional experiments, the sample size was typically at least three, consistent with common practice in the field, with the exception of two experiments performed with two replicates; in those cases, the observed trends were unambiguous.
Data exclusions	Cryo-EM data processing involved removing poor-quality particle images to achieve high-resolution 3D reconstructions through established classification procedures.
Replication	Biochemical experiments were conducted more than three times. Despite some degree of variability in protein yield, the profiles of chromatography were reproducible. Functional experiments were also repeated at least three times with independent samples, with the exception of two experiments performed with two replicates; in those cases, the observed trends were unambiguous.
Randomization	Randomization was not used in this study as the specific types of experiments conducted do not necessitate it; they are not influenced by the sample allocation processes common in other research methods.
Blinding	Blinding was not used in this study as subjective analysis was not needed. Each experiment was analyzed using consistent methods. Quantitative measurements using various approaches as described in the methods minimized biased assessment.

Reporting for specific materials, systems and methods

We require information from authors about some types of materials, experimental systems and methods used in many studies. Here, indicate whether each material, system or method listed is relevant to your study. If you are not sure if a list item applies to your research, read the appropriate section before selecting a response.

Materials & experimental systems

n/a	Involved in the study	
[x]	[ ]	Antibodies
[ ]	[x]	Eukaryotic cell lines
[x]	[ ]	Palaeontology and archaeology
[x]	[ ]	Animals and other organisms
[x]	[ ]	Clinical data
[x]	[ ]	Dual use research of concern
[x]	[ ]	Plants

Methods

n/a	Involved in the study	
[x]	[ ]	ChIP-seq
[x]	[ ]	Flow cytometry
[x]	[ ]	MRI-based neuroimaging

Eukaryotic cell lines

Policy information about cell lines and Sex and Gender in Research

Cell line source(s)	HEK293 (ATCC, CRL-1573) , HEK-293S GnTI– (ATCC, CRL-3022), Sf9 (CRL-1711).
Authentication	No further authentication was performed for commercially available cell lines.
Mycoplasma contamination	Periodically test negative.
Commonly misidentified lines (See ICLAC register)	None of the cell lines used is listed in the database of commonly misidentified cell lines maintained by ICLAC.

Plants

Seed stocks	N/A
Novel plant genotypes	N/A
Authentication	N/A
